# Directly Filtering the Sparse-View CT Images by BM3D

**Published:** 2022-10-07

**Authors:** Gengsheng L Zeng

**Affiliations:** 1 Department of Computer Science, Utah Valley University, USA; 2 Department of Radiology and Imaging Sciences, University of Utah, USA

**Keywords:** Artifacts, Image processing, Image reconstruction, Biomedical imaging, Computed Tomography, Filters

## Abstract

The x-ray Computed Tomography (CT) images with sparse-view data acquisition contain severe angular aliasing artifacts. The common denoising filters do not work well if they are used to reduce the artifacts. The state-of-the-art methods to process the sparse-view CT images are deep-learning based; they require a large amount of training data pairs. This paper considers a situation where no clinical training data sets are available. All we have is one sparse scan of a patient. This paper attempts to use a BM3D filter to reduce the artifacts by using an artifact power spectral density function, which is calculated with computer simulations. The results in this paper show that the proposed method is promising in computer simulations. The proposed method has been applied to patient data, and we observe that the sparse-view artifacts are reduced, especially in the central region of the image, but the artifact reduction is not as effective at the peripheral if the control parameter in the BM3D filter is not properly chosen.

## INTRODUCTION

The motivation for using low-dose x-ray Computed Tomography (CT) is to reduce the patient radiation exposure [1–3]. Since x-ray radiation exposure may play a role in getting cancers, it is advised to reduce the x-ray exposure to an As-Low-As-Reasonably-Achievable (ALARA) level [4]. One way of low-dose imaging is the sparse-view method, but sparse angular sampling frequently leads to characteristic streak artifacts. These artifacts sometimes are referred to as the angular aliasing artifacts [5]. We do not consider other methods of low-dose CT scans such as reducing the X-ray tube current [6–8]. This under sampling situation is also a case of compressed sensing [9,10]. The drawbacks of using reduced radiation exposure include increased noise and artifacts in the CT images. Before reduced radiation dose is used in clinical scans, noise and artifacts must be controlled at the level as if the regular dose is used. The goal of this paper is to investigate whether the sparse-view artifacts can be managed through filtering.

Many researchers attempted to solve this compressed sensing problem. One method is the iterative image reconstruction method that minimizes the Total-Variation (TV) norm or other measures of the image [11–17]. Most recently, research activities are mainly in the deep learning area [18–25]. It is fair to say that deep learning methods are dominating the current publications and conferences. In general, it is unsuccessful to use a denoising filter to combat the artifacts, because the artifacts are shift variant and have certain patterns. There are countless linear and nonlinear filters for noise control. In this paper, we use the term ‘filter’ for an image processing procedure. The application of the hardware X-ray filters (e.g., filters containing Cu or Sn) is not in the scope of this paper.

This paper investigates a nonlinear filter that is not deep learning based. Our filter is based on the BM3D denoising method, which was proposed by Dabov et al. [26,27]. The consideration of the BM3D filter in this paper is motivated by the fact that the BM3D filter is currently the state-of-the-art in terms of noise control. The BM3D method uses block matching and aggregation strategies to obtain three-dimensional image blocks; its denoising uses Wiener filtering. The BM3D method requires two inputs: the noisy raw image and the noise power spectral density image. The original purpose of BM3D is for random noise reduction. In our application of sparse-view tomography, our main concern is the angular aliasing streak artifacts. These artifact patterns are deterministic and object dependent. Artifacts are not random noise. These artifacts are usually more pronounced than the random noise. The strategy of this paper is to treat the deterministic artifacts as random noise when calculating the ‘noise’ power spectral density function (image).

## METHODS

### ‘Noise’ power spectral density

For a given CT image, *G*, resulted from sparse-view projection measurements, its associated artifact power spectral density function, *P*, is difficult to obtain. This is because the true image, *T*, is not available. In this paper, the artifact power spectral density function, *P*, is estimated by noiseless computer simulations, which simulate true image and reconstruction from sparse-scan projections of some random objects. The true images are treated as the gold standard images, *T*_*simu*_.

The artifact image, *A*, is the differences between the gold standard true image, *T*_*simu*_, and the given sparse-scan image, *G*_*simu*_:

(1) $$A=T_{\mathrm{sim}u}-G_{\mathrm{sim}u}.$$

In this paper, we use 1000 random simulated objects. Therefore, we have 1000 two-dimensional artifact images, *A*’s.

Let *B* be the two-dimensional Fourier transform of image *A* defined in Equation (1). For each element in *B*, we calculate its squared norm and denote the resulting frequency-domain image as *P*. This resultant two-dimensional image, *P*, has the same dimension as the image *A*, is real, and is nonnegative. Even in the noiseless cases, *P* is not zero due to the sparse-view streaking artifacts. In forming 1000 versions of *P*, no noise is added. Therefore, the image *P* is better referred to as the artifact spectral function (instead of the noise spectral function).

Let $\bar{P}$ be the average artifact power spectral density image from our 1000 artifact power spectral density images, *P*’s. This averaged artifact power spectral density image $\bar{P}$ is used in the proposed algorithm.

### The proposed algorithm

In the conventional BM3D algorithm, the noise is assumed to be stationary. The Wiener filter is used for denoising in the BM3D algorithm. The Wiener filter assumes stationary noise with a noise power spectral density function $\bar{P}$. However, the artifacts are not stationary. Strictly speaking, it is not proper to use our artifact power spectral density function in the BM3D algorithm. Despite of these concerns, we propose an *ad hoc* algorithm:

(2) $$H=BM3D\left(G_{CT},\bar{P}\right)$$

where *G*_*CT*_ is a two-dimensional given sparse-view CT image, $\bar{P}$ is the averaged artifact spectral density image estimated by computer simulations, *H* is the processed output image, and *BM3D* is the conventional BM3D algorithm.

We must point out that in calculating $\bar{P}$, the sparse simulation *G*_*simu*_ in Equation (1) must have the same imaging and sampling parameters as the situation when sparse-scan CT image, *G*_*CT*_, is obtained. For example, if *G*_*CT*_ is reconstructed from a data set of 180 views and with a focal-point to axis-of-rotation of 600 mm, the *P* image must be obtained using 180 views and 600 mm as well for the sparse-view data. The image reconstruction algorithm must also to be identical for both the computer simulated data and the patient data.

Some notations are summarized in Table 1 in the order of their appearance in the paper.

### Computer simulations

We generated 1000 noiseless random 512×512 phantoms, each of which had two random ellipses of random shapes, random locations, and random intensities. We generated a sparse scan for each phantom: 180 views evenly distributed over 360°. Images were reconstructed using the Filtered Backprojection (FBP) algorithm. One averaged artifact power spectral density image, $\bar{P}$, was calculated from these 1000 phantoms.

We then generated another set of new random 512×512 phantoms and generated corresponding sparse scans with 180 views (test case). The testing phantoms had three random ellipses. Therefore, the testing phantoms and the learning phantoms are different. The FBP reconstruction, *G*_*CT*_, was calculated from the new test case 180-view data. The proposed algorithm (2) was then applied to the FBP reconstruction, *G*_*CT*_, and to obtain the final image, *H*.

### Clinical data

The clinical data used in this paper was acquired from a cadaver for research purposes. It was a full-body scan with 1200 views per rotation. A subset of the projection data was used in our study in the paper. The subset had 180 views evenly distributed over 360°. The x-ray source trajectory was a circle of radius 600 mm. The detector had 320 rows, the row-height was 0.5 mm, each row had 896 channels, and the fan angle was 49.2°. Slices at the abdomen region is shown in the Results section. In this paragraph we explain the relationship between the computer simulations and the clinical data. The proposed algorithm does not work without the simulations. This is because the BM3D filter requires a power spectrum that carries the information of the angular aliasing artifacts. The artifact pattern can only be created by computer simulations. We would like this pattern to be object independent. Our strategy of creating such a pattern is to use 1000 random ‘learning’ phantoms. We project each phantom to obtain a sparse sinogram and then calculate the FBP reconstruction. The reconstruction contains severe angular aliasing artifacts due to the lack of view angles. The artifacts are extracted from the reconstruction. The average of the artifact power spectra from the 1000 random phantoms is calculated as $\bar{P}$. In a clinical application, the proposed BM3D filter needs two inputs: the reconstruction of the clinical image that suffered from angular aliasing artifacts and the artifact power spectral density image $\bar{P}$ estimated from the 1000 random phantom simulations.

## RESULTS AND DISCUSSION

### Computer simulation results

**Figure 1:**
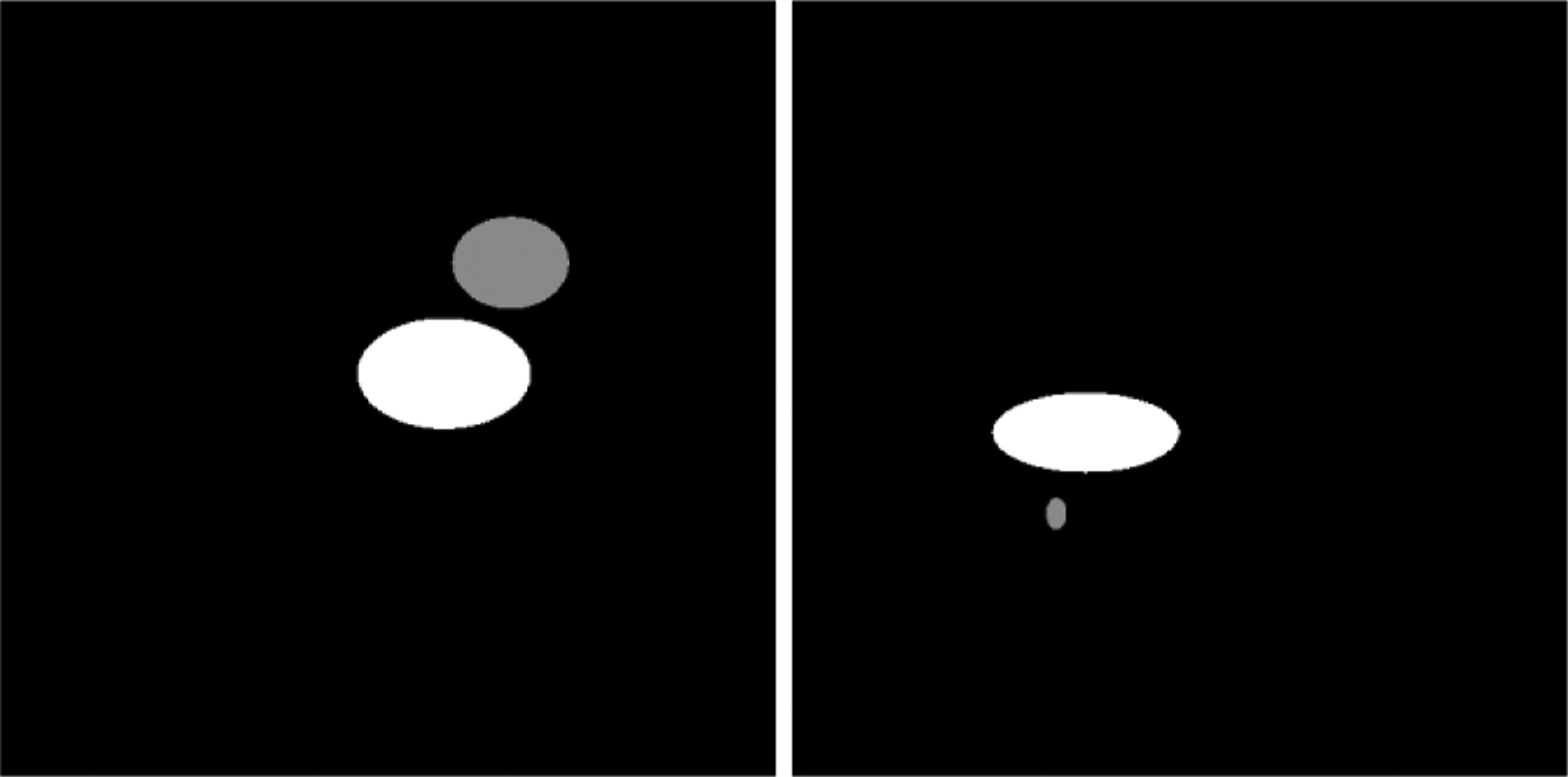
The first two of the 1000 computer simulated random true images. These phantoms are referred to as the learning phantoms. Each random phantom contains two ellipses of random sizes and random intensities.

**Figure 2:**
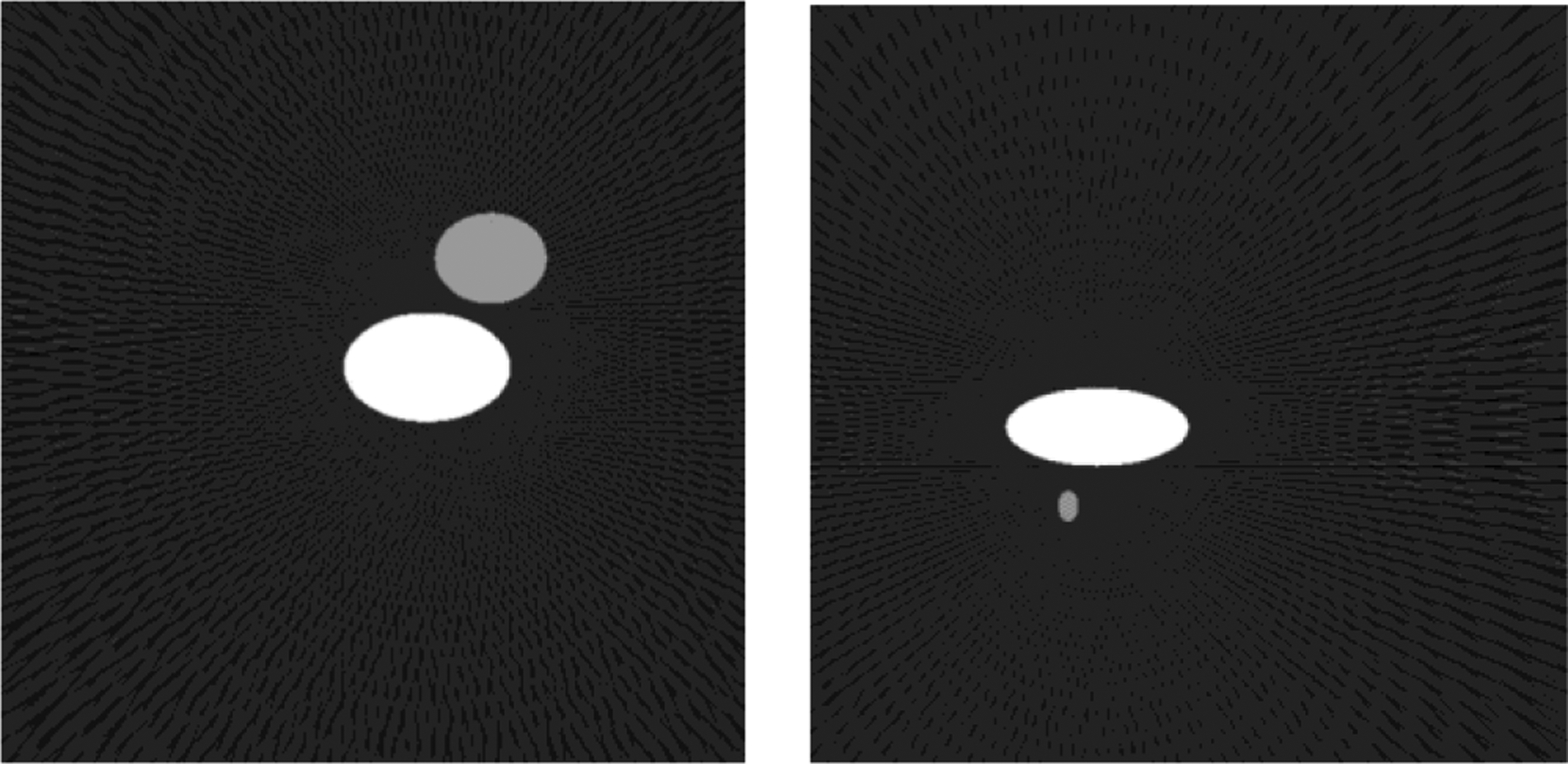
Computer simulated random sparse-scan images corresponding to images in Figure 1. Streaking artifacts are observed. The ARTIFACTS are due the lack of angular measurements.

**Figure 4:**
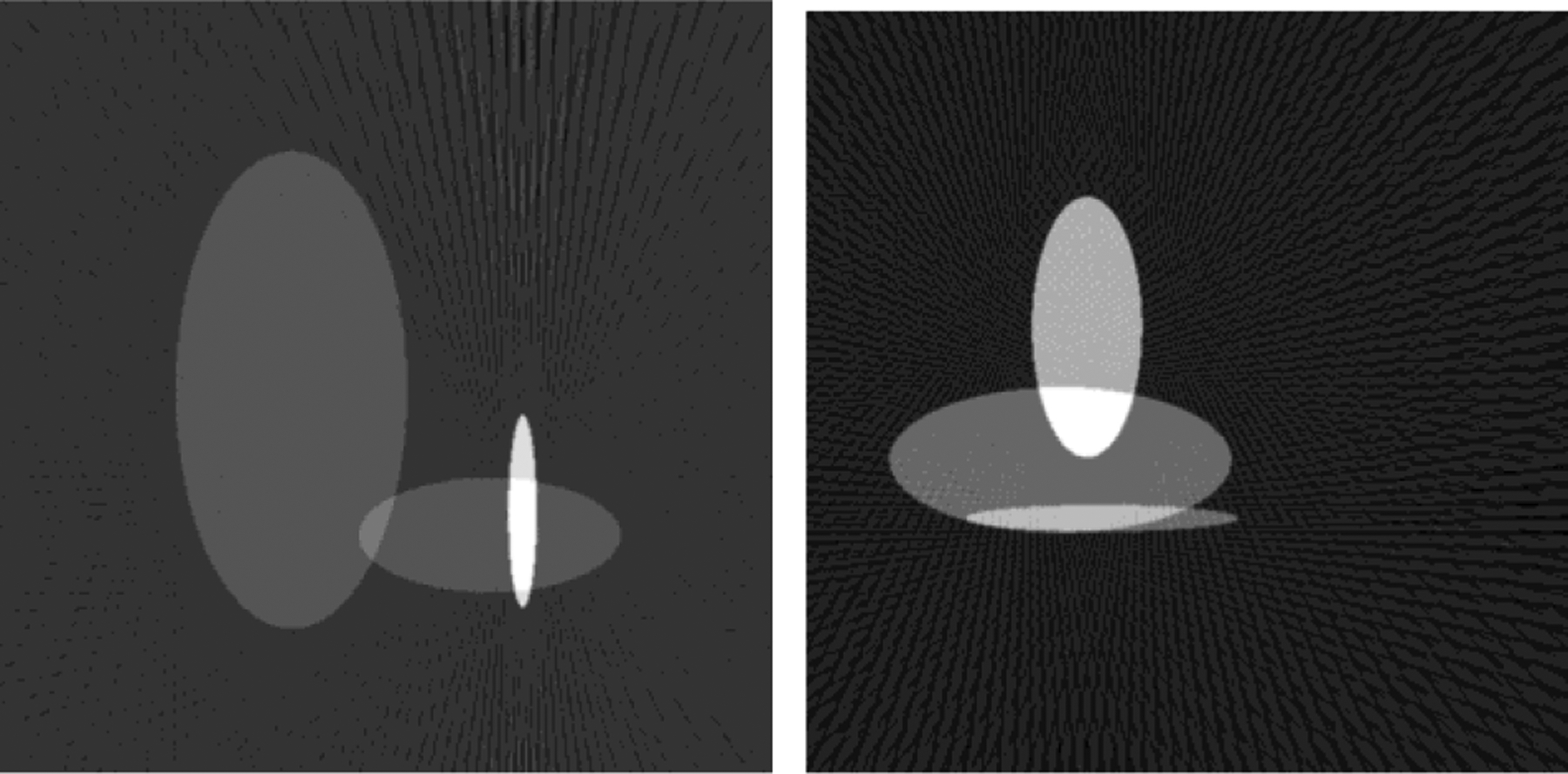
The testing sparse-scan images, which contain three ellipses and are not included in the 1000 random phantoms. Angular aliasing artifacts are observed.

Figure 1 shows the first two of the 1000 random ‘learning’ phantoms. Their sparse-view data reconstructions versions using 180 views are shown in Figure 2, where some angular aliasing artifacts can be visualized in the background. Figure shows the average artifact power spectral density image by considering 1000 sparse/true pairs of the simulated images. Two new random phantom sparse-scan images are shown in Figure 4. These new testing phantoms are NOT among the 1000 learning phantoms used in estimating the artifact power spectral density image, because the new testing phantoms contain three ellipses while the old learning phantoms contain two ellipses. The results of the proposed method are shown in Figure 5, where the angular aliasing artifacts in the background are significantly reduced.

**Figure 6:**
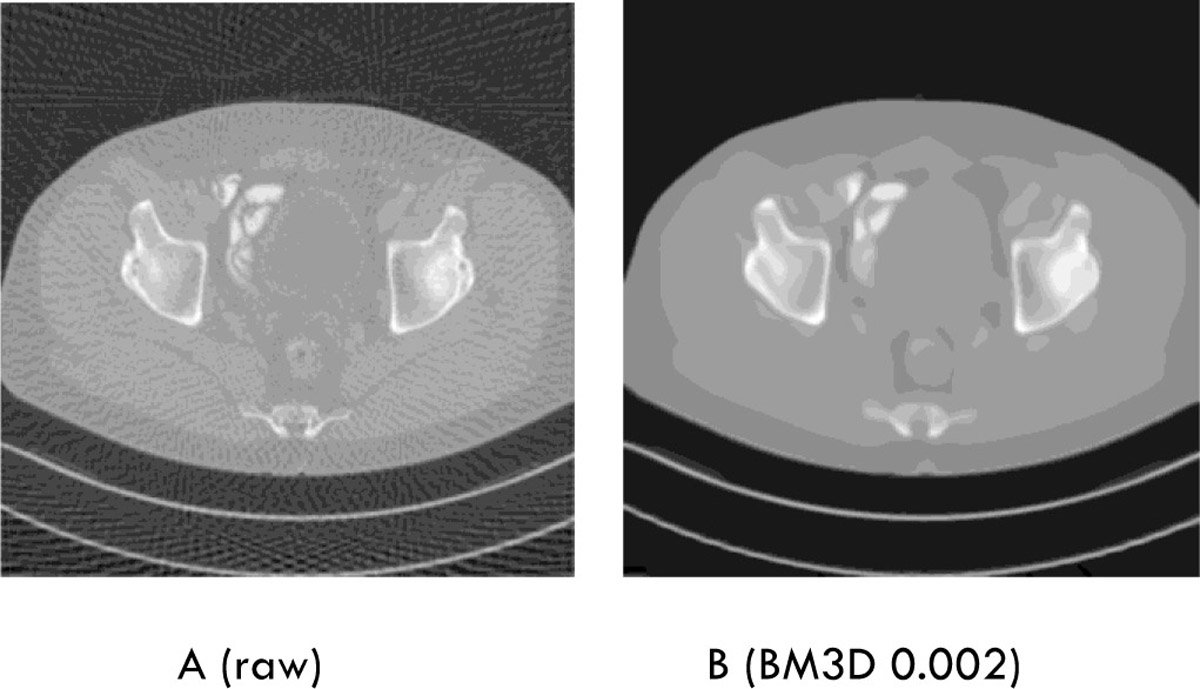

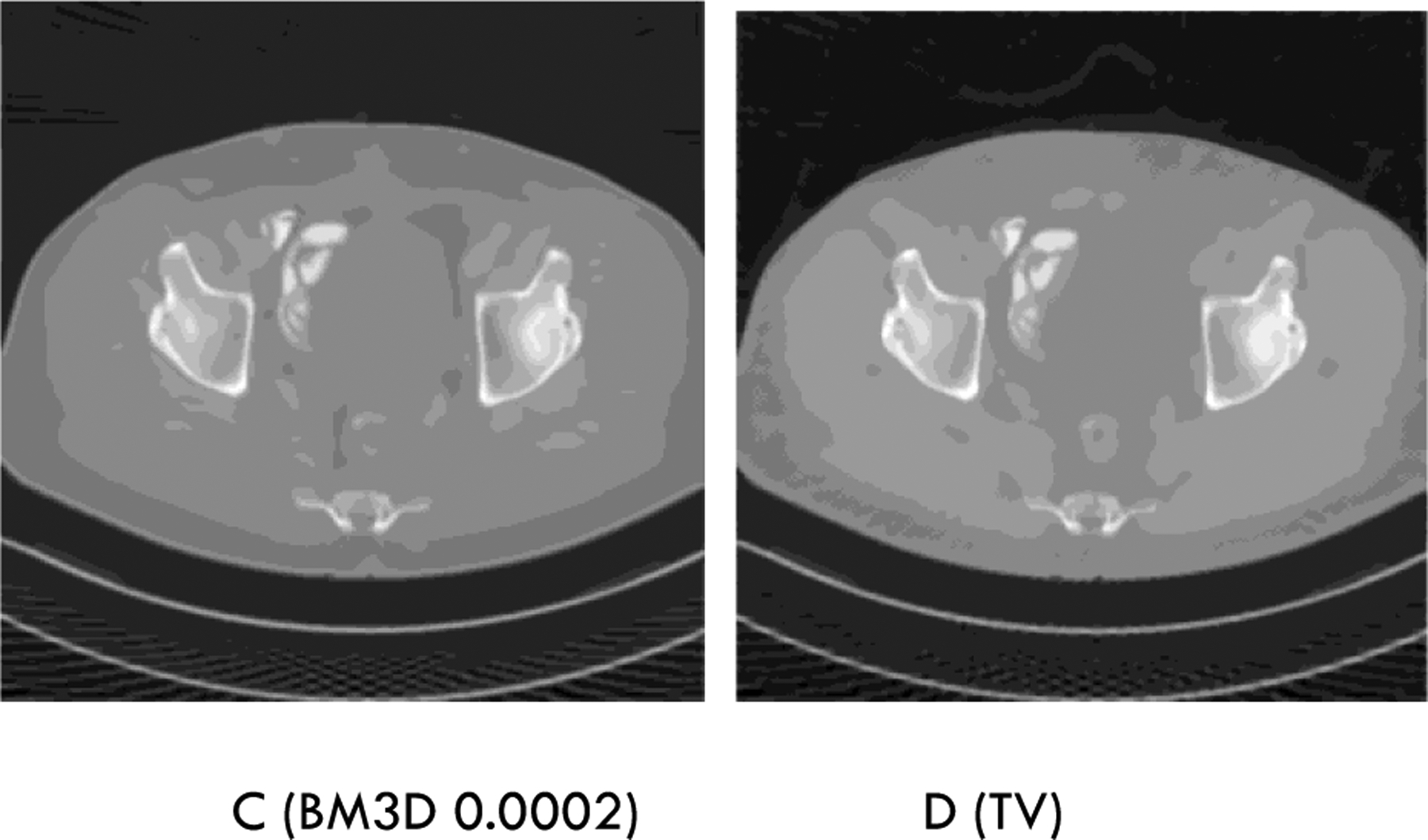
[Patient #1] The sparse-scan patient image slice #160: (A) without processing, (B) with proposed BM3D filter using parameter 0.002, (C) with proposed BM3D filter using parameter 0.0002, and (D) with TV filter.

**Figure 7:**
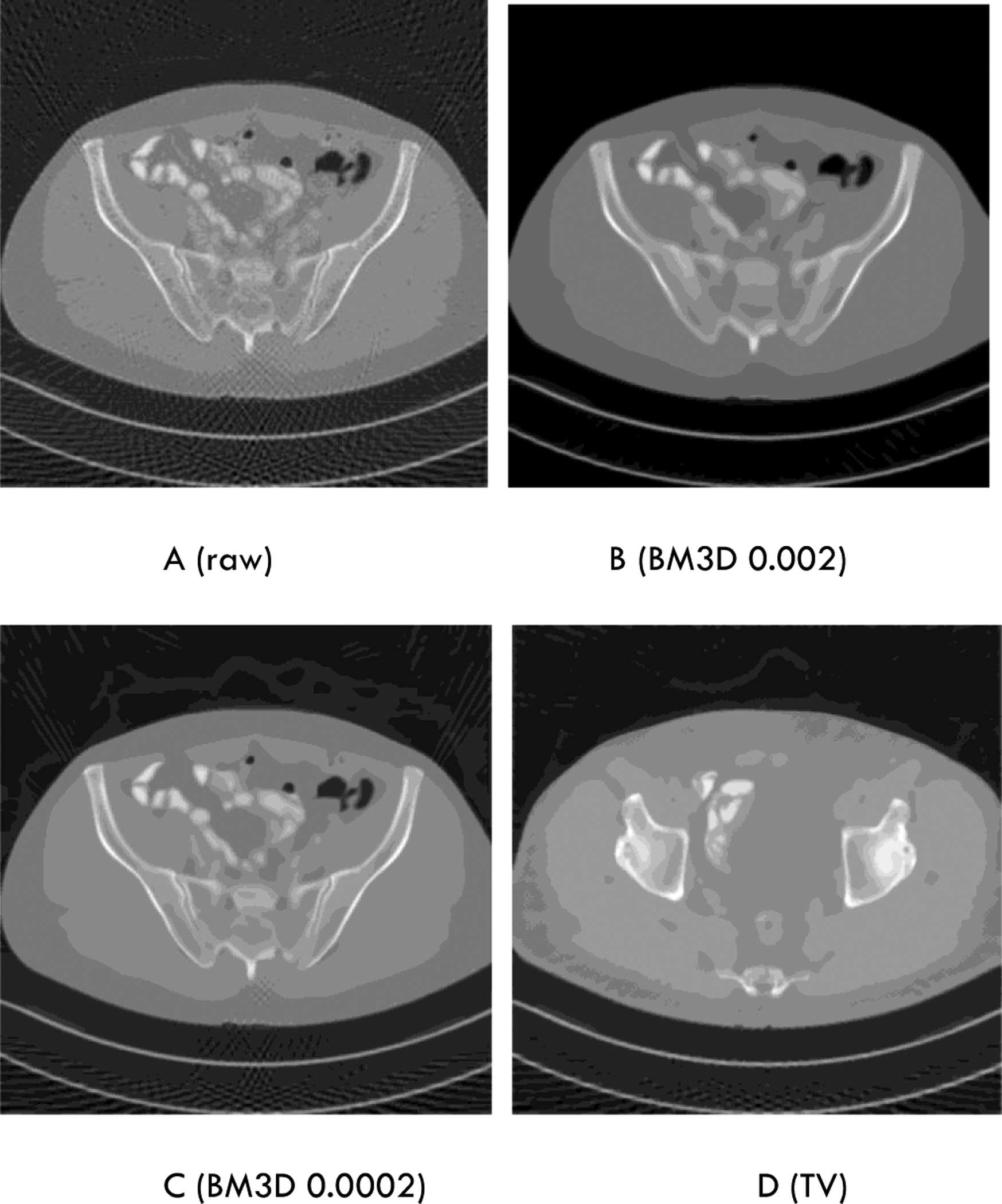
[Patient #1] The sparse-scan patient image slice #140: (A) without processing, (B) with proposed BM3D filter using parameter 0.002, (C) with proposed BM3D filter using parameter 0.0002, and (D) with TV filter.

**Figure 8:**
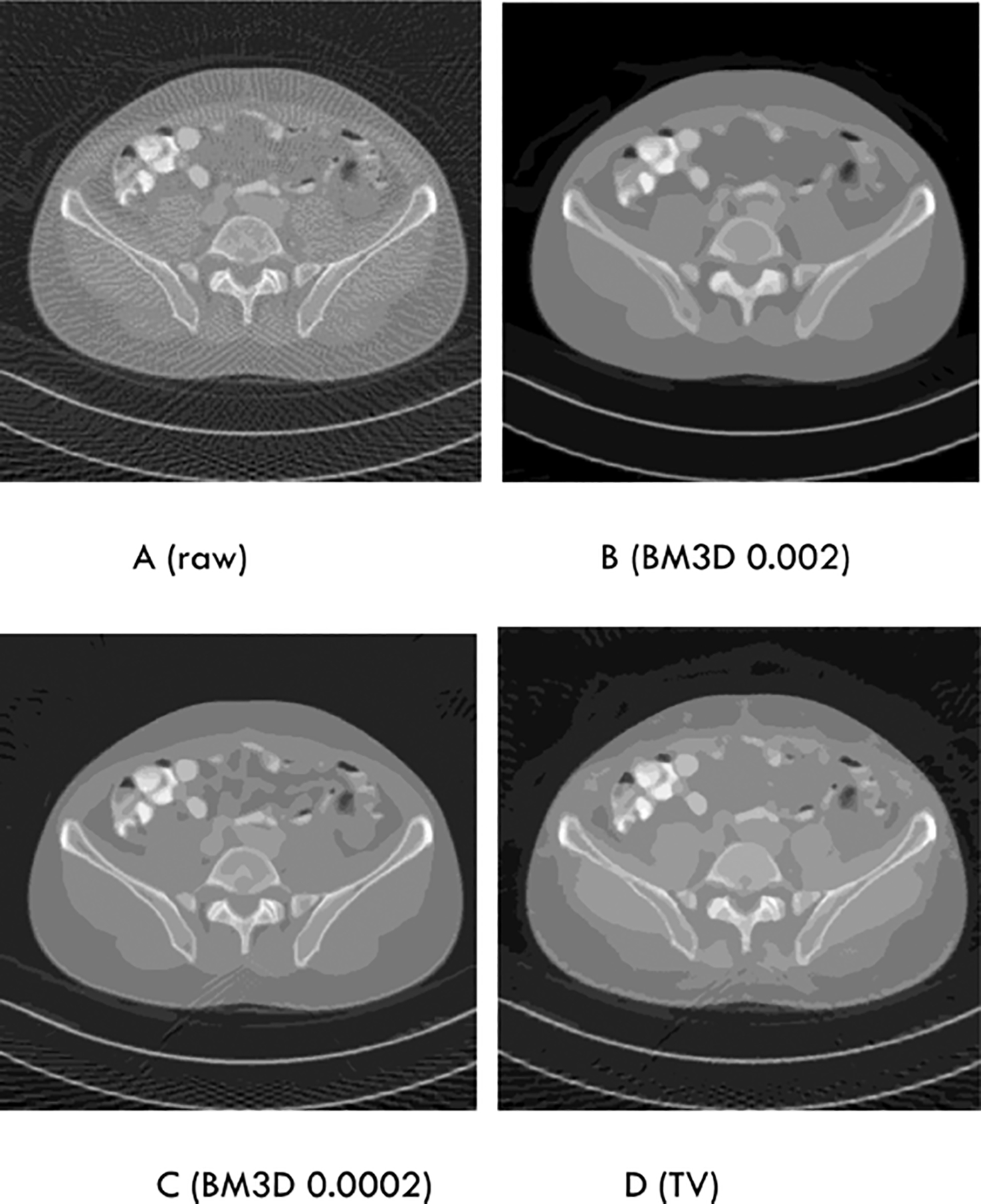
[Patient #1] The sparse-scan patient image slice #120: (A) without processing, (B) with proposed BM3D filter using parameter 0.002, (C) with proposed BM3D filter using parameter 0.0002, and (D) with TV filter.

**Figure 9:**
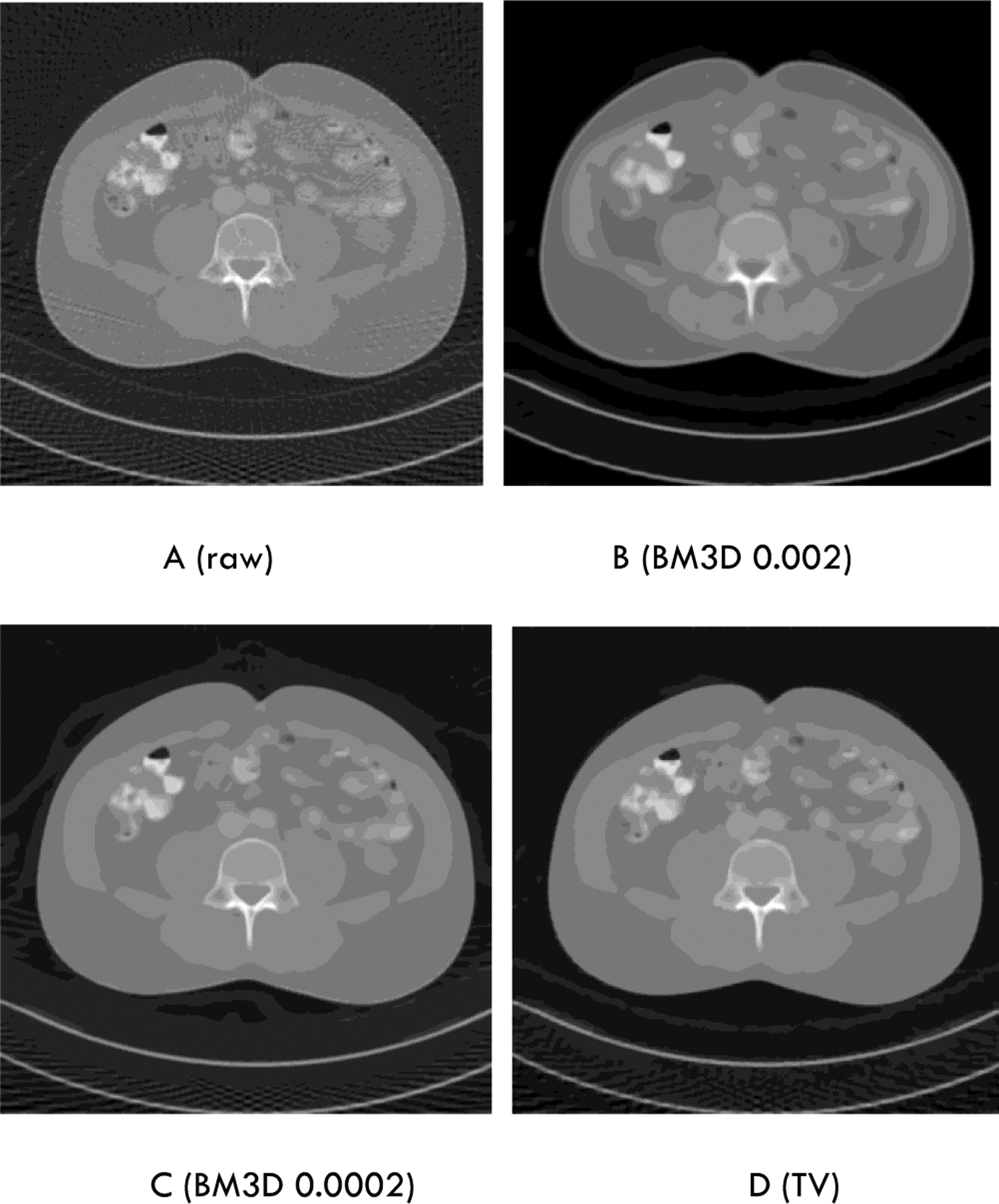
[Patient #1] The sparse-scan patient image slice #100: (A) without processing, (B) with proposed BM3D filter using parameter 0.002, (C) with proposed BM3D filter using parameter 0.0002, and (D) with TV filter.

**Figure 10:**
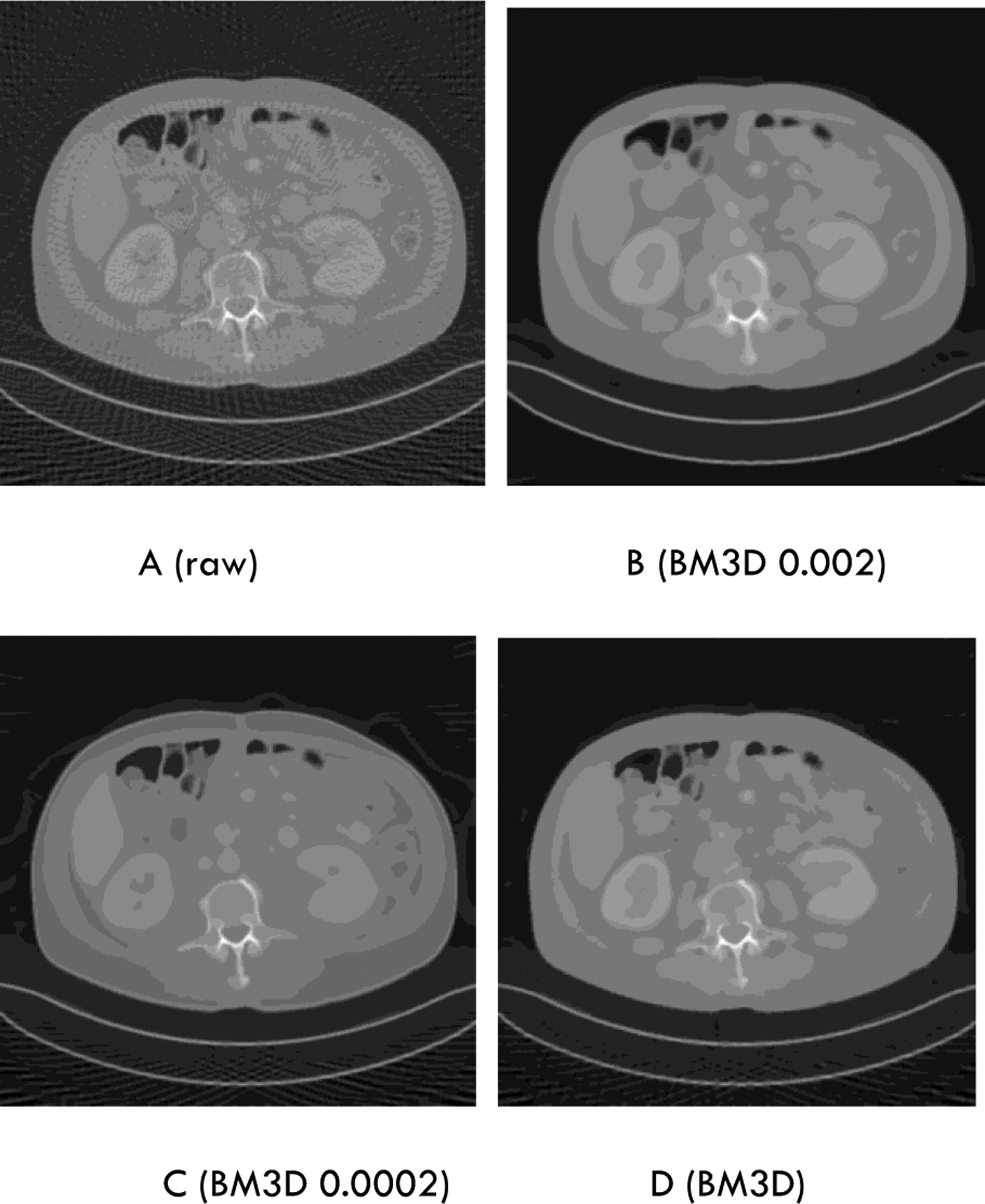
[Patient #2] The sparse-scan patient image slice #80: (A) without processing, (B) with proposed BM3D filter using parameter 0.002, (C) with proposed BM3D filter using parameter 0.0002, and (D) with TV filter.

**Figure 11:**
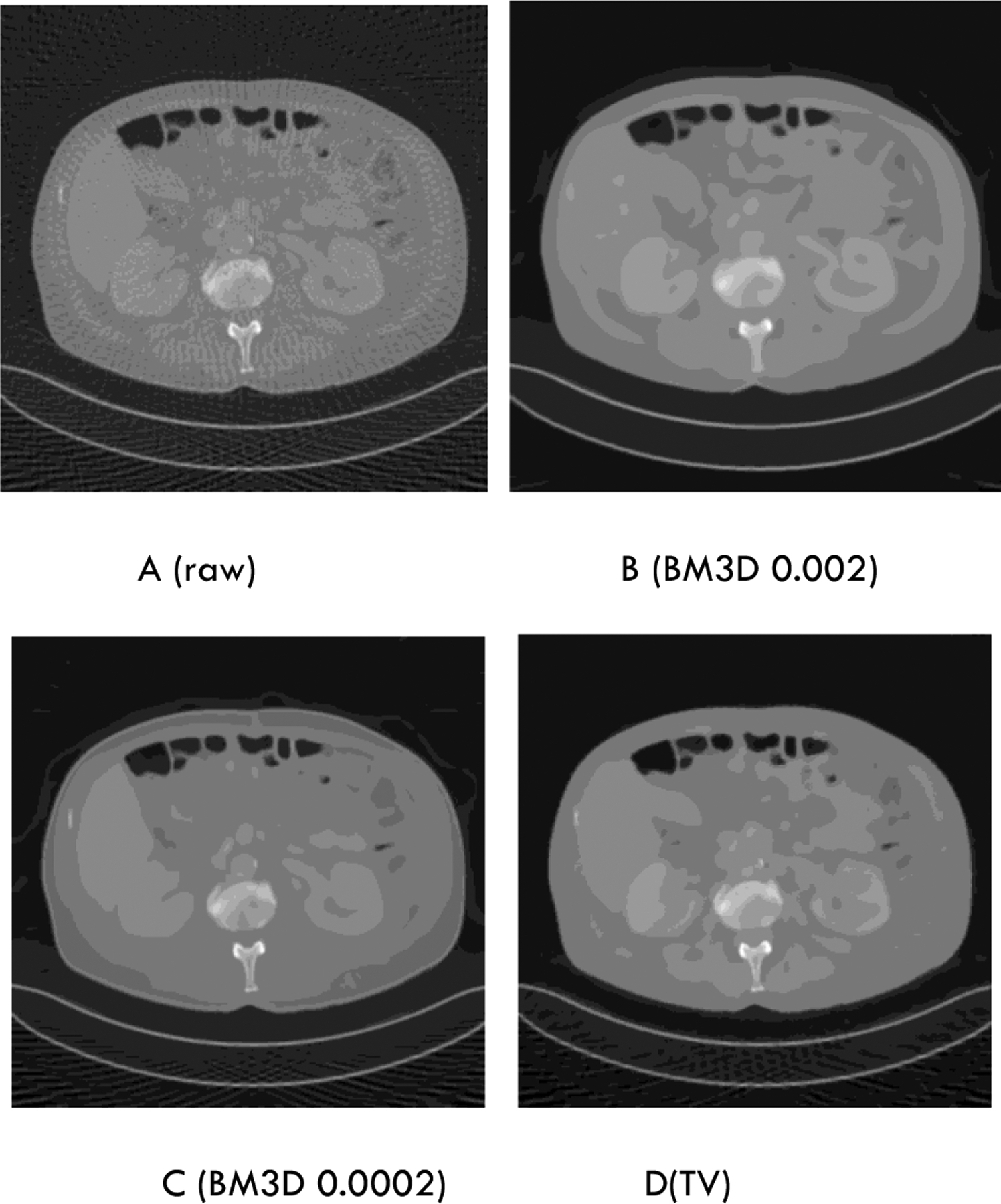
[Patient #2] The sparse-scan patient image slice #70: (A) without processing, (B) with proposed BM3D filter using parameter 0.002, (C) with proposed BM3D filter using parameter 0.0002, and (D) with TV filter.

**Figure 12:**
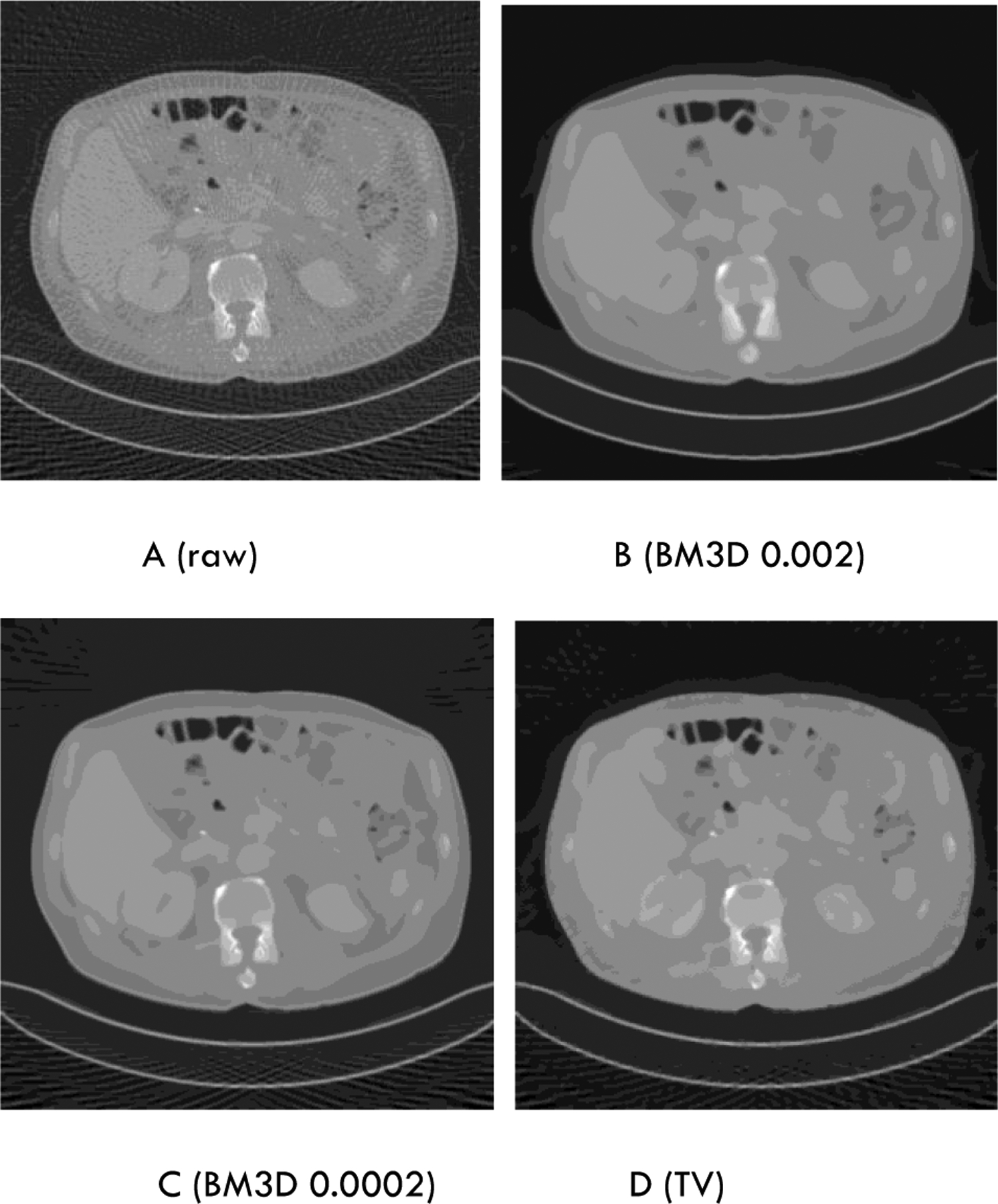
[Patient #2] The sparse-scan patient image slice #60: (A) without processing, (B) with proposed BM3D filter using parameter 0.002, (C) with proposed BM3D filter using parameter 0.0002, and (D) with TV filter.

**Figure 13:**
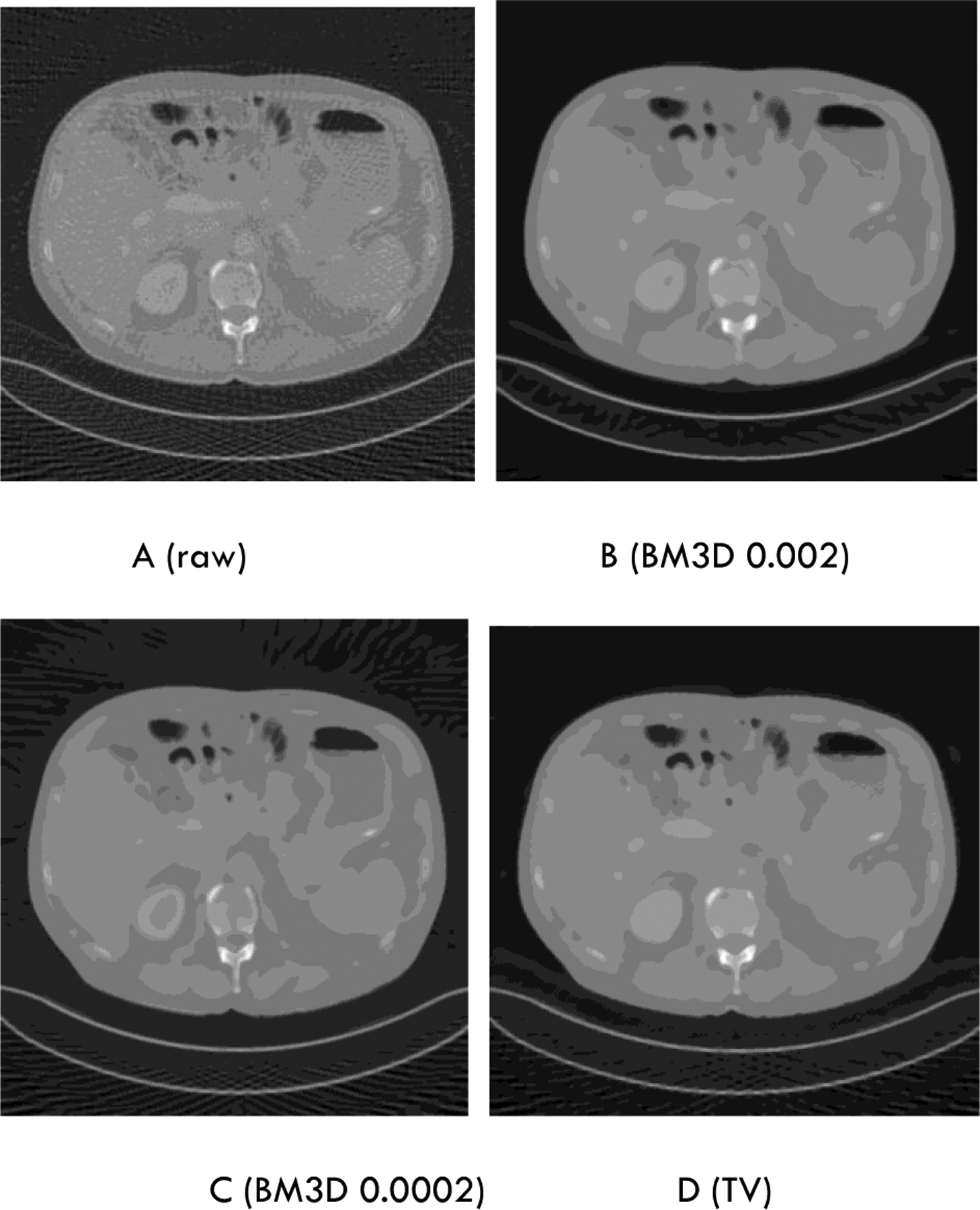
[Patient #2] The sparse-scan patient image slice #50: (A) without processing, (B) with proposed BM3D filter using parameter 0.002, (C) with proposed BM3D filter using parameter 0.0002, and (D) with TV filter.

Eight patient image pairs from two different patients are shown in (Figure 6–13), respectively. The images are sparse-scan images without and with the proposed BM3D processing. It is observed that the proposed algorithm is partially successful in reducing the angular aliasing artifacts. The proposed algorithm is more effective in the central region of the image. Each of the CT patient study has many different slices. Data from two different patients are used. Results from slices 100, 120, 140, and 160 of the first patient and results from slices 50, 60, 70, and 80 are presented. The main drawback of the proposed method is that the BM3D filter tends to over smooth the images. This drawback is also observed for the TV method. The BM3D method has a user selected hyper parameter. If the parameter is not properly chosen, the image may be either over smoothed or artifacts are not removed. How to select this hyper parameter is *ad hoc*. Two selections of this parameter are used in the patient data processing: 0.002 and 0.0002. The parameter of 0.002 in the BM3D filter makes the image over smoothed. However, the artifacts are still visible if the parameter of 0.0002 is used in the BM3D filter.

The purpose of the computer simulations with 1000 random phantoms is to generate an artifact power spectrum image $\bar{P}$. The first two of the 1000 computer simulated random true images are shown in Figure 1. Each random phantom contains ellipses of random sizes and random intensities. A sinogram is generated with 180 views evenly distributed over 360°, and then the FBP algorithm is used for images reconstruction. It is seen in Figure 2 that the reconstructions contain the streaking angular aliasing artifacts. No noise is added to the projections. The artifacts are caused by the imaging geometry. The metric of numerical evaluation is the matrix L2 norm of the difference image between the true image and the FBP reconstruction is calculated. The numerical evaluation results are listed in Table 2 for the computer simulations, and in Table 3 for the clinical data studies. An iterative Total-Variation (TV) norm minimization algorithm is implemented and compared with the BM3D method. These two methods show comparable performances. The drawback of these two algorithms is that they depend on some hyper parameters. The hyper parameters are chosen ad hoc. If the parameters are not properly chosen, the resultant image may be over smoothed or may still contain the artifacts.

## DISCUSSION AND CONCLUSIONS

**Figure 14:**
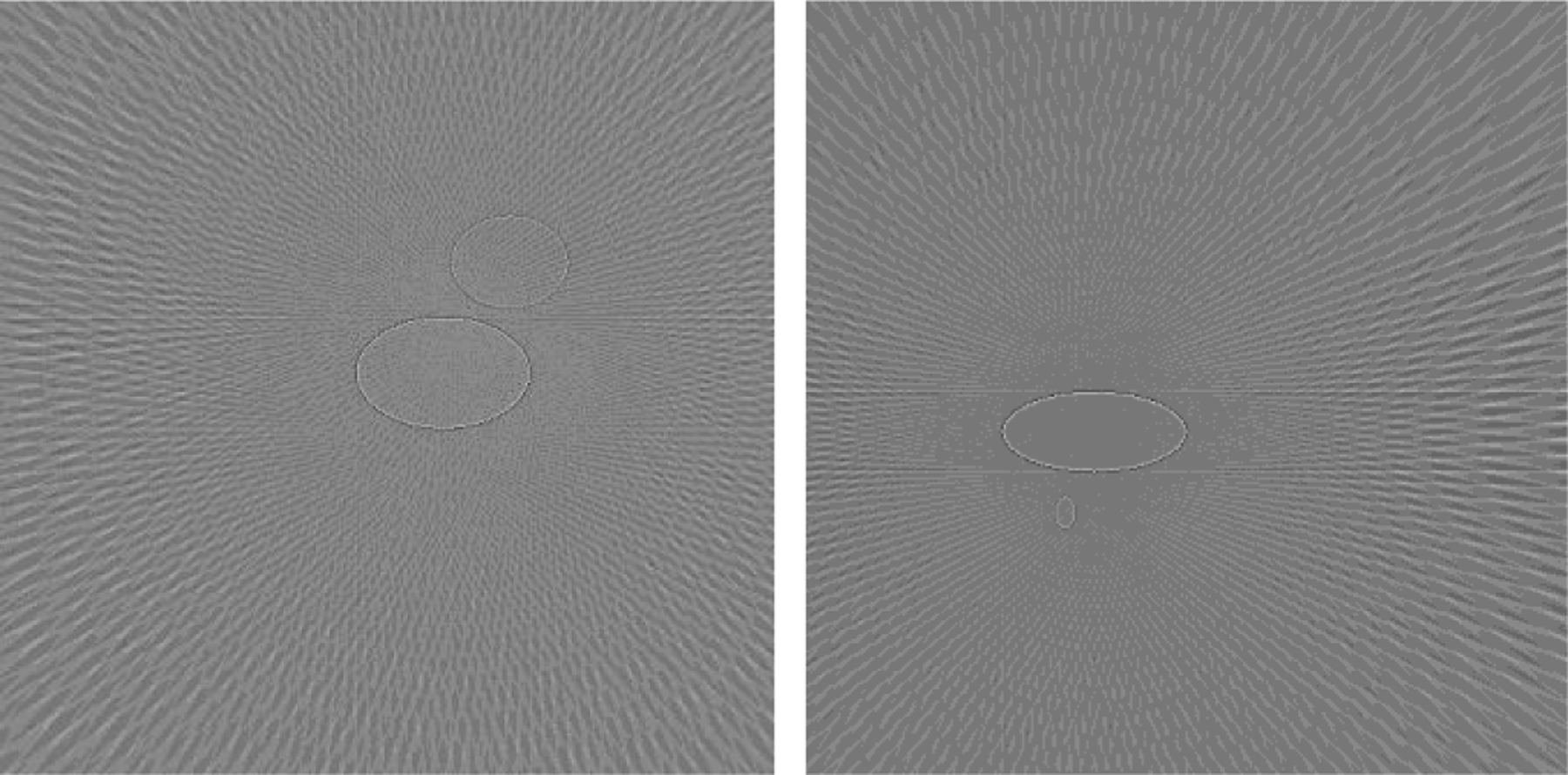
One example of artifact images calculated for the computer simulated random phantoms.

The innovative idea in this paper is to replace the noise power spectral density image by the artifact power spectral density image. The artifact power spectral density image is estimated by 1000 computer simulated random phantoms. The random learning phantoms only contain two ellipses and do not look like human torsos at all. Unlike the common practice in machine learning, where the training data are very similar to the testing data. Why do we need as many as 1000 random images to estimate the artifact power spectral density image? To answer this question, let us look at Figure 14, where two artifact images calculated by (1) are shown. The artifact images contain artifacts also contain some shape information of the random phantoms. We do not want the phantom information leak into the artifact power spectral density image. By using the average of a large number of the artifact power spectral density images, the influence of the phantom shape information can be significantly reduced.

We have attempted a method to reduce the sparse-scan angular aliasing artifacts without using any patient training data. This method is a direct application of the BM3D filter by replacing the noise power spectral density function with the artifact power spectral density function. On the other hand, the method of using a point source to estimate the Point Spread Function (PSF) does not work. This is because the angular aliasing artifacts are object size dependent. The required number of views in tomography data acquisition is proportional to the diameter of the object. The artifact creation is not a linear and shift-invariant phenomenon. A point source can be exactly reconstructed by using only two views. The BM3D filter assumes stationary noise that is characterized by the noise power spectral density function. Noise and artifacts are never the same. Noise is random, while artifacts are somewhat deterministic. Artifacts are not stationary. Strictly speaking, the artifact power spectral density function does not exist because it is not stationary.

Our *ad hoc* method assumes the norm square of the Fourier transform of the error image as the artifact power spectral density function, which is calculated with computer simulations and depends on the imaging geometry only. Patient data is not used in finding the artifact power spectral density function. Our results indicate that the proposed method is not yet effective enough for practical applications. If the hyper parameter in BM3D filter is not properly chosen, the artifacts are still present, and the images are over-smoothed after processing. More work needs to be done. However, insights our from this study suggest that some features can be obtained by simulations when there is no real data available. Another thing we observe is that the Wiener filter is not an effective method to remove artifacts, and a better approach should be considered.

## Figures and Tables

**Figure 3:**
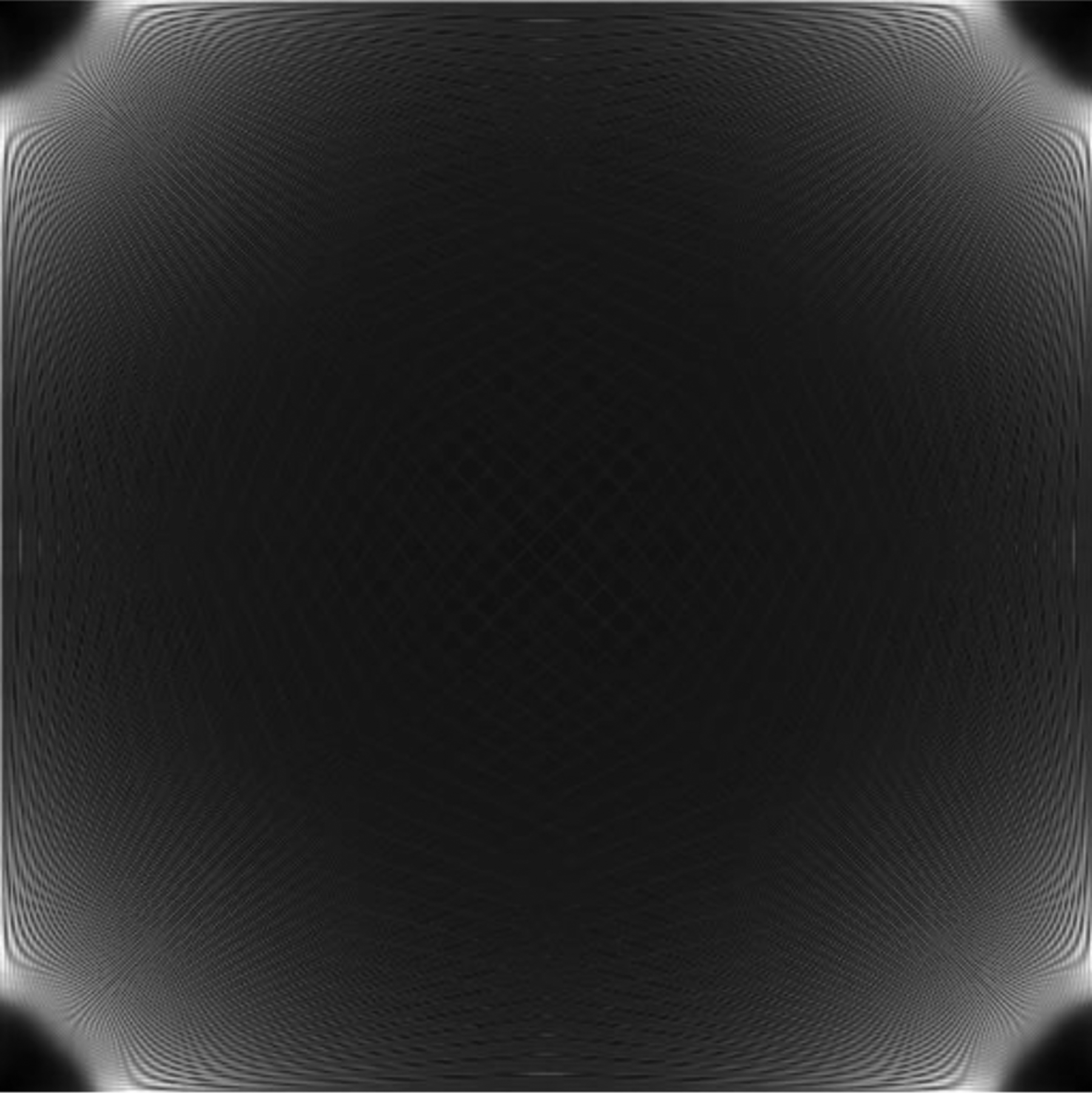
The averaged artifact power spectral density image for the computer simulation study corresponding to images in Figure 1. The power spectral density image is computed in the Fourier domain. The center of the image is the zero frequency. The corners are the high frequencies.

**Figure 5A.**
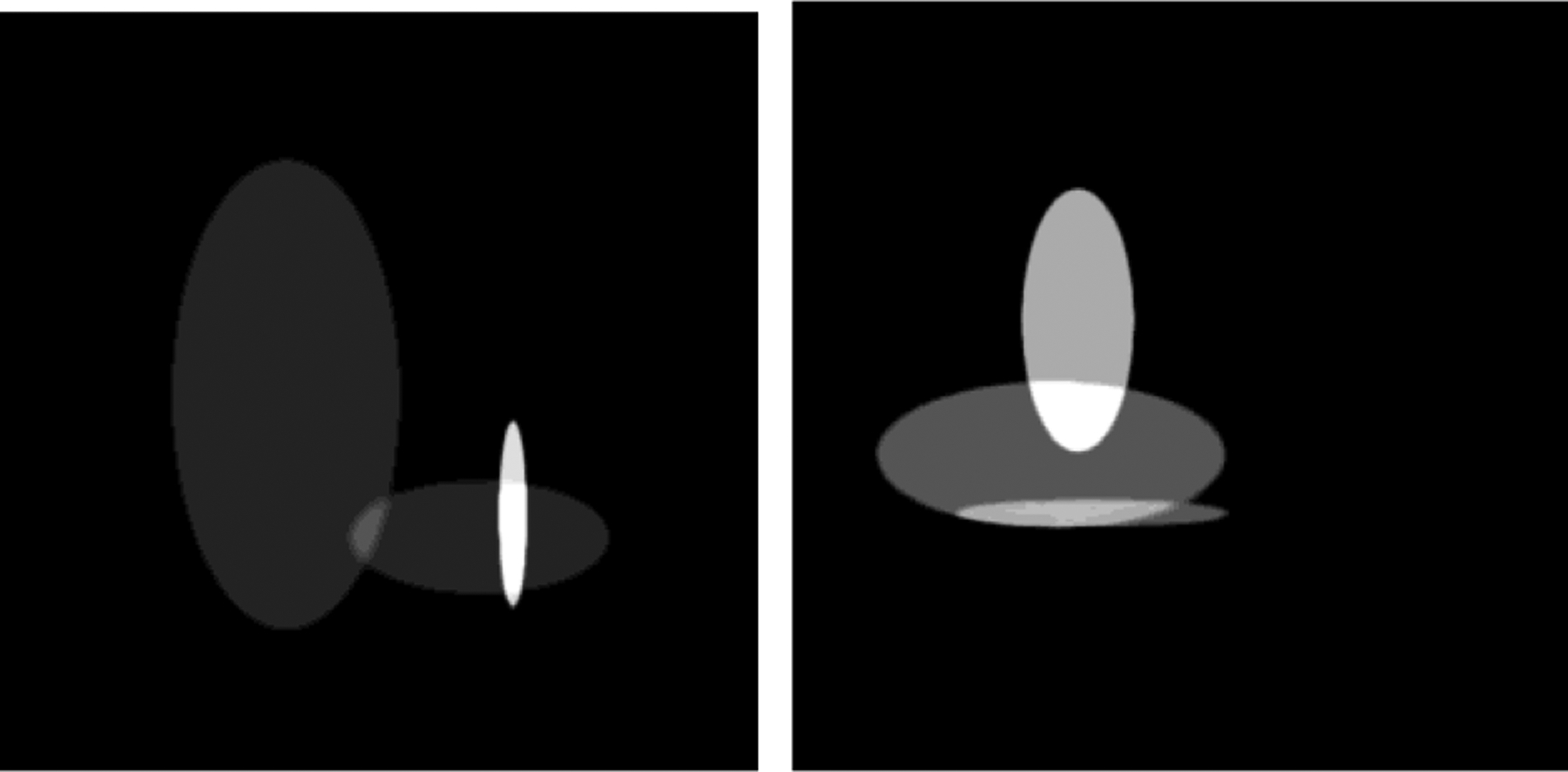
The testing sparse-scan images processed by the proposed method, corresponding to the images in Figure 4. The angular aliasing streaking artifacts are essentially removed.

**Figure 5B:**
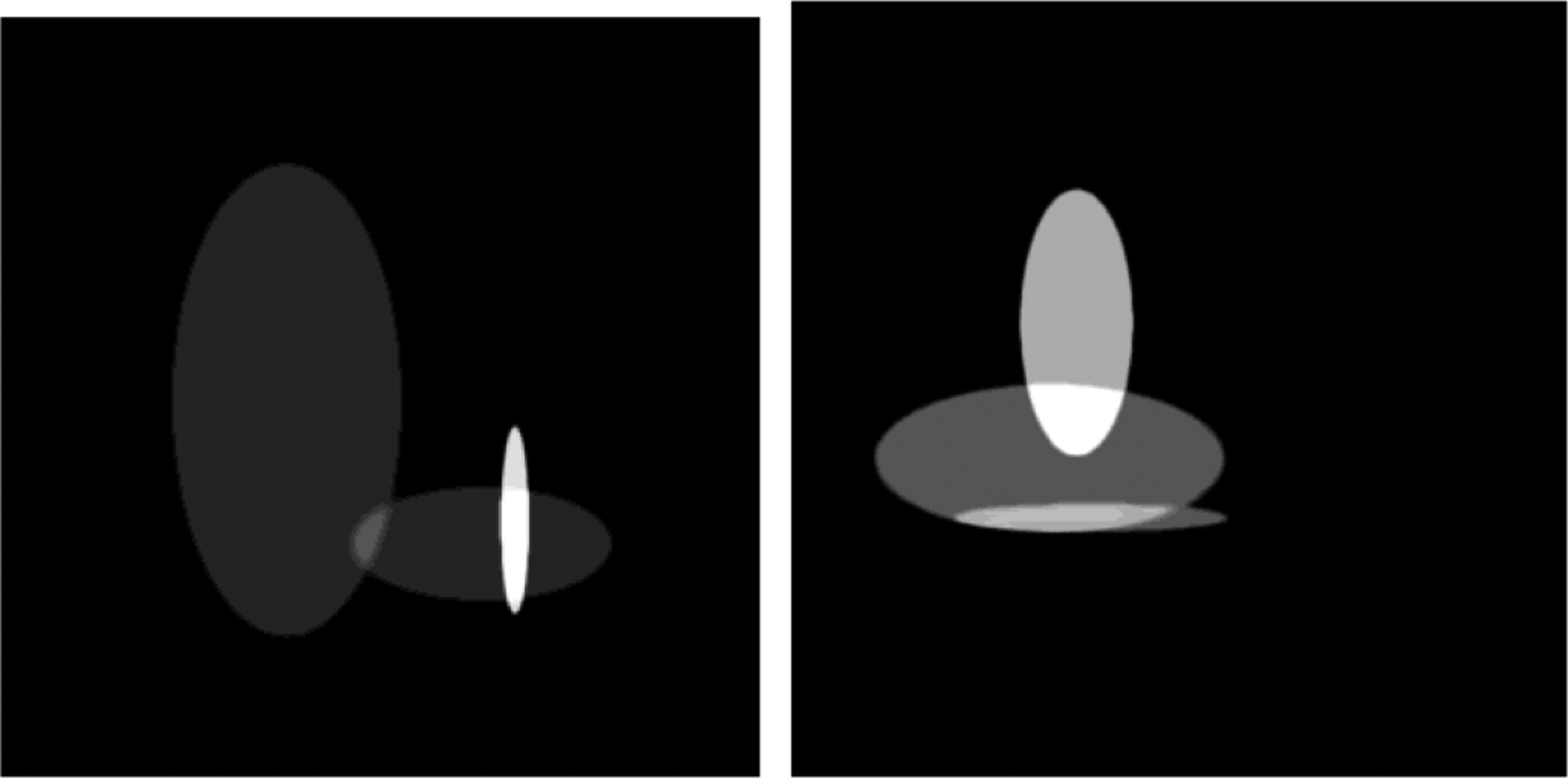
The testing sparse-scan images processed by the TV method, corresponding to the images in Figure 4.

**Table 1:** Designations and their definitions.

Designation	Definition
*T_simu_*	True image
*G_simu_*	Sparse-scan image
*A*	Artifact image
*B*	Fourier transform of image *A*
*P*	Element-by-element squared norm of *B*; artifact power spectral image
*P*	Averaged artifact power spectral density image
*G_cr_*	A two-dimensional given sparse-view CT image to be processed
*H*	The output image of BM3D

**Table 2:** Mean squared error of different artifact removal methods using computer simulated data.

Phantom	Raw	Proposed method	TV method
1	4.1836	2.0298	2.9412
2	3.4784	1.8826	3.8068
3	3.3696	1.7359	2.3718
4	7.3443	1.6206	7.5332
5	3.9122	1.4504	4.1005
6	3.7584	1.3857	3.9307
7	14.3733	2.5038	14.4774
8	5.1276	1.9214	5.4700
9	5.4813	1.8168	5.7350
10	7.4323	1.6374	7.5671

**Table 3:** Mean squared error of different artifact removal methods using clinical data.

Slice number	Raw	Proposed method (0.002)	Proposed method (0.0002)	TV method
100 (patient #1)	346.79	5.0650	12.899	8.1203
120 (patient #1)	360.40	3.6040	13.001	8.1825
140 (patient #1)	382.79	4.8907	12.810	7.7631
160 (patient #1)	386.75	4.4841	11.729	8.1034
50 (patient #1)	359.45	4.2961	9.8627	7.0827
60 (patient #1)	355.34	4.5427	10.181	7.3332
70 (patient #1)	349.22	4.5455	9.8969	7.0459
80 (patient #1)	343.58	4.4938	9.9216	7.0175

## References

[1] National Council on Radiation Protection and Measurements. (2009). NCRP Report No. 160: Ionizing Radiation Exposure of the Population of the United States. 142–146.

[2] Lell M, Kachelrieß M. (2020). Recent and upcoming technological developments in computed tomography: High speed, low dose, deep learning, multienergy. Investigative Radiology. 55: 8–19. 31567618 10.1097/RLI.0000000000000601

[3] Messerli M, Kluckert T, Knitel M, Walti S, Desbiolles L, (2017). Ultralow dose CT for pulmonary nodule detection with chest x-ray equivalent dose — a prospective intra-individual comparative study. Eur Radiol. 27: 3290–3299. 28093625 10.1007/s00330-017-4739-6

[4] Yeung WK. (2019). The ‘As Low As Reasonably Achievable’ (ALARA) principle: a brief historical overview and a bibliometric analysis of the most cited publications. Radioprotection. 54: 103–109.

[5] Kak C, Slaney M. (1988). Principles of Computerized Tomographic Imaging. IEEE Press.

[6] Zeng GL. (2020). “Pre-filter that incorporates the noise model,” Visual Computing for Industry. Biomedicine and Art 3. 13. 10.1186/s42492-020-00051-z PMC7242545 32440712

[7] Zeng GL. (2020). Fast filtered back projection algorithm for low-dose computed tomography,” Journal of Radiology and Imaging. 4: 45–50. 10.14312/2399-8172.2020-7 PMC8294203 34295998

[8] Zeng GL. (2022). Photon starvation artifact reduction by shift-variant processing. IEEE. 10: 13633–13649. 10.1109/access.2022.3142775 PMC9390879 35993039

[9] Chen H, Zhang Y, Chen Y, Zhang J, Zhang W, (2018). LEARN: Learned Experts’ Assessment-Based Reconstruction Network for Sparse-Data CT,” IEEE Transactions on Medical Imaging. 37: 1333–1347. 29870363 10.1109/TMI.2018.2805692 PMC6019143

[10] Donati L, Nilchian M, Trépout S, Messaoudi C, Marco S, (2017). Compressed sensing for STEM tomography. Ultramicroscopy. 179: 47–56. 28411510 10.1016/j.ultramic.2017.04.003

[11] Sidky EY, Pan X. (2008). Image reconstruction in circular cone-beam computed tomography by constrained, total-variation minimization. Phys Med Biol. 53: 4777–4807. 18701771 10.1088/0031-9155/53/17/021 PMC2630711

[12] Abbas S, Min J, Cho S. (2013). Super-sparsely view-sampled cone-beam CT by incorporating prior data. J X-Ray Sci Technol. 21: 71–83. 10.3233/XST-130367 23507853

[13] Huang J, Zhang Y, Ma J, Zeng D, Bian Z, (2013). Iterative image reconstruction for sparse-view CT using normal-dose image induced total variation prior. PLoS One. 8: e79709. 24260288 10.1371/journal.pone.0079709 PMC3832537

[14] Zheng Z, Hu Y, Cai A, Zhang W, Li J, (2019). Few-view computed tomography image reconstruction using mean curvature model with curvature smoothing and surface fitting. IEEE Trans Nucl Sci. 66: 585–596.

[15] Jones GA, Huthwaite P. (2018). Limited view X-ray tomography for dimensional measurements. NDT & E Int. 93: 98–109.

[16] Vlasov VV, Konovalov AB, Kolchugin SV. (2018). Hybrid algorithm for few-views computed tomography of strongly absorbing media: algebraic reconstruction, TV-regularization, and adaptive segmentation. J. Electron Imag 27: 043006.

[17] de Molina, Serrano E, Garcia-Blas J, Carretero J, Desco M, (2018). GPU-accelerated iterative reconstruction for limited-data tomography in CBCT systems. BMC Bioinformatics. 19: 171. 29764362 10.1186/s12859-018-2169-3 PMC5952580

[18] Han Y, Ye JC. (2018). Framing U-net via deep convolutional framelets: application to sparse-view CT. IEEE Transactions on Medical Imaging. 37: 1418–1429. 29870370 10.1109/TMI.2018.2823768

[19] Lee H, Lee J, Kim H, Cho B, Cho S. (2019). Deep-neural-network-based sinogram synthesis for sparse-view CT image reconstruction. IEEE Transactions on Radiation and Plasma Medical Sciences. 3: 109–119.

[20] Zhang Z, Liang X, Dong X, Xie Y, Cao G. (2018). A sparse-view CT reconstruction method based on combination of DenseNet and deconvolution. IEEE Transactions on Medical Imaging. 37: 1407–1417. 29870369 10.1109/TMI.2018.2823338

[21] Xie S, Zheng X, Chen Y, (2018). Artifact removal using improved GoogleNet for sparse-view CT reconstruction. Sci Rep. 8: 6700. 29712978 10.1038/s41598-018-25153-w PMC5928081

[22] Wu W, Hu D, Niu C, Yu H, Vardhanabhuti V, (2021). DRONE: Dual-domain residual-based optimization network for sparse-view CT reconstruction. IEEE Transactions on Medical Imaging. 40: 3002–3014. 33956627 10.1109/TMI.2021.3078067 PMC8591633

[23] Zhang, Li Y, Chen G-H. (2021). Accurate and robust sparse-view angle CT image reconstruction using deep learning and prior image constrained compressed sensing (DL-PICCS). Medical Physics. 48: 5765–5781. 34458996 10.1002/mp.15183 PMC9580024

[24] Liu J, Sun Y, Gan W, Xu X, Wohlberg B, (2021). SGD-Net: Efficient model-based deep learning with theoretical guarantees. IEEE Transactions on Computational Imaging. 7: 598–610.

[25] Xiang J, Dong Y, Yang Y. (2021). FISTA-net: Learning a fast iterative shrinkage thresholding network for inverse problems in imaging,” IEEE Transactions on Medical Imaging. 40: 1329–1339. 33493113 10.1109/TMI.2021.3054167

[26] Dabov K, Foi A, Katkovnik V, Egiazarian K. (2007). Image denoising by sparse 3-D transform-domain collaborative filtering. IEEE Trans. Image Process 16: 2080–2095. 17688213 10.1109/tip.2007.901238

[27] Dabov K, Foi A, Katkovnik V, Egiazarian KO. (2008). Image restoration by sparse 3D transform-domain collaborative filtering. Image Processing: Algorithms and Systems VI, International Society for Optics and Photonics; San Jose, CA, USA. 6812: 1–7.

